# Using Health data to analyse repeat victimisation in Scotland.

**DOI:** 10.23889/ijpds.v7i3.1820

**Published:** 2022-08-25

**Authors:** Ana Morales, Susan McVie

**Affiliations:** 1 University of Edinburgh

## Objectives

The aim of this project is to analyse temporal patterns of repeat violent victimisation (RVV) in Scotland using administrative data and explore the extent to which these patterns are associated with underlying health vulnerabilities (related to mental health and problematic alcohol and/or drug use) and vary by individual socio-demographic characteristics.

## Approach

Violence in Scotland, and especially RVV, is a ‘key public health priority’. However, very little is known about the link between violence and other health vulnerabilities at an individual level, which may be useful in informing prevention approaches. This paper uses data on violence-related ambulance call-outs linked to hospital records containing information on wider health vulnerabilities. We use survival mixture models to identify and characterise population sub-groups, based on time to repeated violent episodes and explore how different health-related factors are associated with RVV and how this varies across sub-populations.

## Results

We identify two classes of RVV with two distinct survival trajectories: a ‘low vulnerability’ class and a ‘high vulnerability’ class. People in the high vulnerability class experienced RVV more quickly and were more likely to have underlying mental health conditions and/or problematic drug or alcohol use than those in the low vulnerability class. The association between socio-demographic characteristics and RVV differed across the two classes, with significant inequality in RVV for high-risk individuals between those in the most and the least affluent communities. The findings suggest that the first 6 months after the index event is a critical period of RVV for people in the high-risk group, but there may be different exposure mechanisms for different types of victims.

## Conclusion

Better use of administrative data from health sources could help improve our understanding and identification of repeat victims of violence. The ability to identify whether victims are likely to be ‘high-risk’ or ‘low-risk’ could inform the design of focused strategic approaches and interventions aimed at reducing repeat violent victimization.

